# Stabilizing and strengthening the US physician-scientist faculty workforce in academic medicine: a proposed institutional framework

**DOI:** 10.1172/jci.insight.205939

**Published:** 2026-04-28

**Authors:** Christopher S. Williams, Megan Allen, Paige Cooper-Byas, John Hawley, Louis J. Muglia, E. Dale Abel, Julie A. Bastarache, Carolyn Calfee, John M. Carethers, David N. Cornfield, Oliver Eickelberg, Emily J. Gallagher, Anna Greka, Peter J. Gruber, Anthony N. Hollenberg, Heidi H. Kong, Barbara Kazmierczak, Gary A. Koretzky, Mark Lachs, Deborah J. Lenschow, Geoffrey Pitt, Don C. Rockey, Lisa M. Satlin, Barry P. Sleckman, David A. Stoltz, Jatin M. Vyas, Thomas J. Wang, Kyu Y. Rhee

**Affiliations:** 1 Division of Gastroenterology, Department of Medicine, Vanderbilt University Medical Center, Nashville, Tennessee, USA.; 2 Damon Runyon Cancer Research Foundation, New York, New York, USA.; 3 Burroughs Wellcome Fund, Research Triangle Park, North Carolina, USA.; 4 American Society for Clinical Investigation, Ann Arbor, Michigan, USA.; 5 Department of Medicine, David Geffen School of Medicine, UCLA, Los Angeles, California, USA.; 6 Division of Allergy, Pulmonary, and Critical Care Medicine, Vanderbilt University Medical Center, Nashville, Tennessee, USA.; 7 Division of Pulmonary, Critical Care, Allergy, and Sleep Medicine, Department of Medicine, UCSF, San Francisco, California, USA.; 8 Department of Medicine, Moores Cancer Center, and Herbert Wertheim School of Public Health and Human Longevity, UCSD, San Diego, California, USA.; 9 Division of Pulmonary, Asthma, and Sleep Medicine, Department of Pediatrics, School of Medicine, Stanford University, Stanford, California, USA.; 10 Division of Pulmonary, Allergy, Critical Care, and Sleep Medicine, University of Pittsburgh School of Medicine, Pittsburgh, Pennsylvania, USA.; 11 Division of Endocrinology, Diabetes and Bone Disease, Department of Medicine, Icahn School of Medicine at Mount Sinai, New York, New York, USA.; 12 Broad Institute of MIT and Harvard, Cambridge, Massachusetts, USA.; 13 Division of Cardiac Surgery, Department of Surgery, Yale School of Medicine, New Haven, Connecticut, USA.; 14 Department of Medicine, Boston Medical Center and Boston University Chobanian and Avedisian School of Medicine, Boston, Massachusetts, USA.; 15 Chevy Chase, Maryland, USA.; 16 Departments of Medicine and Microbial Pathogenesis, Yale School of Medicine, New Haven, Connecticut, USA.; 17 Office of the Provost, Cornell University, Ithaca, New York, USA.; 18 Division of Geriatrics and Palliative Care Medicine, Weill Cornell Medicine, New York, New York, USA.; 19 Department of Medicine, Washington University School of Medicine, St. Louis, Missouri, USA.; 20 Cardiovascular Research Institute, Weill Cornell Medicine, New York, New York, USA.; 21 Digestive Disease Research Center, Medical University of South Carolina, Charleston, South Carolina, USA.; 22 Department of Pediatrics, Icahn School of Medicine at Mount Sinai, New York, USA.; 23 Department of Medicine, Heersink School of Medicine, O’Neal Comprehensive Cancer Center, University of Alabama at Birmingham, Birmingham, Alabama, USA.; 24 Department of Internal Medicine, University of Iowa, Iowa, USA.; 25 Division of Infectious Disease, Department of Medicine, Columbia University Vagelos College of Physicians and Surgeons, New York, New York, USA.; 26 University of Michigan Medical School, Ann Arbor, Michigan, USA.; 27 Department of Medicine, Weill Cornell Medicine, New York, New York, USA.

## Abstract

Physician-scientists represent one of the most impactful, yet underrecognized, innovations of 20th century academic medicine. Defined by a commitment to full-time careers in investigative work, physician-scientists have repeatedly demonstrated a unique ability to identify and solve problems of unmet medical need in a focused and intentional manner using their dual training in clinical medicine and the scientific method as both stethoscope and scalpel. Unfortunately, while the value of physician-scientists has never been greater, the institutional infrastructure to support them has never been explicitly defined (1), a deficiency now amplified by mounting financial pressures from both clinical revenue models and an increasingly constrained research funding landscape (2, 3). This white paper reports the output of a consortium of academic medical centers, foundations, and professional societies seeking to remedy this deficiency. This consortium specifically developed a framework to formalize the career path of physician-scientist faculty into a professionally unified and financially sustainable structure amenable to adoption across US academic medical centers and health systems. Key components of this framework included an administratively operational definition of physician-scientists, and 3 central and interconnected pillars (academic, financial, and organizational) that are rooted in this foundational definition. Herein, we detail core concepts and concrete recommendations.

## Abstract 2

This white paper proposes a dedicated framework intended to help academic medical centers and health systems support and maximize their return-on-investment in physician-scientists and the cures of tomorrow.

## Introduction

Trained in both clinical medicine and scientific investigation, physician-scientists have repeatedly demonstrated the unique ability to transform patient-inspired needs into scientifically solvable problems whose solutions improve the health care of tomorrow (4–8). Yet, while leaders in academic medicine recognize the unique value of physician-scientists, the need for dedicated support for the distinctive and specific professional needs of this small, but essential, workforce remains surprisingly unmet (1, 3), resulting in inefficiency, instability, attrition, and most importantly, missed opportunities to improve human health. From a historical perspective, physician-scientists have made groundbreaking contributions from both the public and private sectors that have transformed clinical medicine, as exemplified by their outsized representation among Nobel laureates and leadership positions among the top 10 pharmaceutical companies and the NIH (9). Yet, their representation within the biomedical workforce has steadily declined both in relative and absolute numbers over the past 4 decades by nearly 70% (9). Left unchecked, this decline threatens not only our capacity to advance medicine through scientific discovery but also our ability to capitalize on emerging technologies and therapeutics whose development demands the unique translational insight that only physician-scientists provide.

Notwithstanding critical deficiencies associated with entry into the independent workforce from the training pipeline (10–12), physician-scientists face the broader challenge of navigating a career lacking an aligned vocational structure. That is, the absence of clear metrics and standards for support of physician-scientists (13) has confounded efforts to attract, efficiently train, and retain the next generation of this essential workforce. This report proposes a conceptual framework with the potential to remedy these deficiencies by formalizing the career path into a vocationally distinct and unified structure that can be adapted/scaled to different academic medical centers and health systems. The need for such a structure was prompted by 2 inter-related factors: (a) the historical failure to adequately distinguish the contributions of physician-scientists from those of academic clinicians and non-clinician investigator counterparts (9, 14–16), and (b) the growing inability of existing metric-based business models of clinical care and research to adequately account for the contributions from this hybrid career (17, 18). Developing such a structure would not only furnish a more uniform and stable platform that enables physician-scientists to more predictably realize their potential but would also facilitate the development of more structured onramps to the independent workforce.

Despite their widely recognized contributions to each of the 3 components of the academic mission, physician-scientists lack a clearly articulated vocational identity that is specifically tied to the unique, and inherently evolving, nature of their contributions; a distinction that has been blurred by the shared role of scholarship across the academic mission. In fact, current models used to support physician-scientists remain rooted in academic and financial paradigms that predate the era of multidisciplinary translational research (19) and continue to compartmentalize medicine and science as vocationally distinct careers. Physician-scientists have thus been left to (a) simultaneously straddle part-time clinical and research career paths (13), each of which are associated with an increasingly complex and demanding set of metrics and expectations that are effectively independent of one another, and (b) depend on financial models that were developed to support full-time careers in only one or the other and have become increasingly unforgiving (20). The continued use of these models, which undervalue the unique contributions made by physician-scientists, is incompatible with the viability and fundamental mission of physician-scientists and threatens their ability to integrate science and medicine in a professionally and vocationally aligned manner (3, 10).

**Figure 1:**
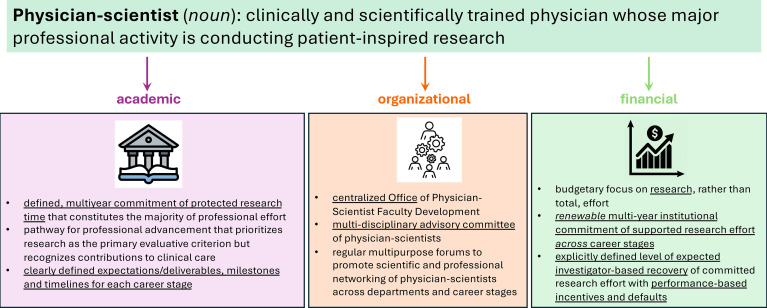
Proposed institutional framework to support the physician-scientist career track. Placeholder.

The framework proposed in this report (Figure 1) reflects the proceedings of a 1.5-day workshop jointly convened by the American Society for Clinical Investigation, the Burroughs Wellcome Fund, the Damon Runyon Cancer Research Foundation, and experts from 18 academic medical centers spanning 14 states inclusive of both public and private institutions. The goal of this workshop was to first develop an operational definition of the physician-scientist that (a) spans the NIH T0–T4 spectrum of translational research (21) — from basic discovery (T0) to early human testing (T1) to clinical trials and guideline development (T2) to clinical implementation (T3) to population-level outcomes (T4) — across different clinical and scientific disciplines; and (b) could be adapted within and across different organizational structures. This definition was then followed by the development of core structural pillars needed to operationalize this definition in practice. We elaborate on specifics of each below.

## Definition and foundational concepts

The proposed framework rests atop the following operational definition of physician-scientists: “clinically and scientifically trained physicians whose major professional activity is conducting patient-inspired research.” This definition is both concrete and differentiates physician-scientists from academic clinicians and non-clinical investigator peers on 2 attributes: (a) their dual training in clinical medicine and scientific research and (b) their explicit engagement in extramural peer-reviewed research as their primary professional activity (22). While the term “physician-scientist” has historically been used to describe physicians performing laboratory-based investigation, and “clinical investigators” as those that conduct various forms of clinical and population health research, the operational definition of physician-scientists proposed here captures the most important and distinguishing functional attributes common to physician-scientists across the translational spectrum while also accommodating field- or discipline-specific differences in their specific activities and recognizing the equal importance of each. From a functional perspective, this definition highlights core structural elements required for success: training, protected research time/committed effort, and resources (1). This definition additionally emphasizes the fundamental importance of their clinical expertise in directing and defining the impact of their research program, rather than as a financial failsafe. This perspective similarly acknowledges both the inadequacy of year-to-year clinical budgets to provide the stability required for the success of research projects, and the irreplaceable necessity of multiyear funding for this purpose. The proposed definition is more concretely tied to 2 linked requirements: an explicit need for clearly defined multiyear commitments of effort and funding and a reciprocal set of equally well-defined expectations matched to these commitments. This definition finally, and perhaps most significantly, meets the practical need of being one that can be administratively defined and tracked based on the basis of committed clinical and research effort and support.

## Structural pillars

Seeking to operationalize the foregoing definition, the workshop identified the need for the following 3 linked and interdependent structural pillars: academic, financial, and organizational. Each was developed and unified with the others through an alignment around the foundational definition described above. Below, we elaborate on fundamental components of each.

### Academic pillar.

Physician-scientists require a distinct academic support structure that explicitly recognizes their intended role as clinically trained full-time investigators focused on solving problems of unmet medical need (3, 22, 23). The absence of such a dedicated structure has historically splintered the physician-scientist workforce across different professional tracks within and across institutions, resulting in a blurring of their identity to both the institution and the investigator. In seeking to remedy this deficiency, the following 4 core elements were identified: (a) an extended/multiyear commitment of protected research time that constitutes the majority of professional effort and is sufficient to establish and maintain an extramurally funded research program (24); (b) a pathway for professional advancement that prioritizes research as the primary evaluative criterion but recognizes the unique contributions of physician-scientists to clinical care and teaching; (c) clearly defined expectations/deliverables and competencies for each career stage, including measurable milestones that recognize the inherently unpredictable outcomes associated with scientific research (25); and (d) formalized mentorship, both scientific and clinical, at every stage from trainee to junior faculty to mid-level and even senior positions that recognizes the evolving, if not expanding, career trajectory of physician-scientists (26).

The specific minimum level of protected research time considered “sufficient to establish and maintain an extramurally funded research program” is recognized to potentially vary based on whether the specific type of research being conducted is laboratory-, patient-, or population-based and should also reflect what is needed to maintain clinical skills. NIH guidance for early-stage physician-scientists transitioning to independence requires a minimum of 75% protected research time, with surgeon- and procedure oriented-scientists requiring a minimum of 50% to accommodate the need to maintain clinical proficiency. For investigators whose research is directly tied to clinical care, or whose clinical activity may require a minimum amount of effort to maintain procedural proficiency, this allocation of clinical effort may suffice but should be inclusive of linked administrative effort that has historically gone unaccounted for. The duration of precommitted protected research time allocated for a given individual may similarly vary based on the specific type and area of research being conducted but should be compatible with prevailing grant funding rates (24) and accommodate any linked and/or required clinical responsibilities.

The establishment of an explicitly defined advancement and/or promotion pathway for physician-scientists is essential to ensure equitable coupling of professional goals to career development. Traditional tenure-track faculty appointment and promotion structures and processes generally lack the ability to recognize or account for the clinical activity of physician-scientists (27), other than as an administrative justification to extend the tenure clock beyond those of non-clinical faculty. Academic clinical tracks conversely emphasize clinical productivity and teaching over research and, in some cases, is further rewarded by financial clinical incentives. For some institutions, the absence of a dedicated pathway has splintered physician-scientists across faculty tracks with potentially competing goals that distract and potentially delay or diminish their ultimate impact. Revisions to existing faculty tracks and/or entirely new tracks that define criteria specific to physician-scientists across career stages thus constitute a critical unmet need at many institutions. The need for such revisions was noted to be particularly acute for physician-scientists engaged in large collaborative “team science”–based initiatives (28) that often make critical contributions and/or play key leadership roles that are often inadequately articulated and/or recognized.

The call for competency and milestone-based expectations recognizes the need to balance career advancement metrics with purpose- or product-focused goals of the physician-scientist. Unlike the case for fundamental scientists whose goal is to generate new knowledge in the absence of a specific medical problem, physician-scientists are positioned to pursue questions directly relevant to human health and disease. As such, it becomes possible, and perhaps even incumbent, for physician-scientists to be able to measure the direction and extent of progress toward the problem of interest. Such metrics similarly apply to physician-scientists engaged in basic science research due to their ability to understand and articulate the potential relevance of their discoveries to human disease, even if not immediately translatable. The standardized development and use of objectively demonstrable, predefined milestones of progress, based on career stage and scientific complexity, offers an opportunity to evaluate an investigator along the path to achieving their ultimate goal(s). Setting milestones additionally creates the ability and opportunity to evaluate and demonstrate the specific value of physician-scientists to institutions within and outside of academia.

While mentorship is an essential component of all careers, it is of heightened importance to physician-scientists because of their dual activities in clinical medicine and scientific research and hybrid identities that inherently evolve over time. Physician-scientists thus require a panel of mentors that are capable of not only providing domain-specific expertise but also integrating them in a manner that aligns their professional focus with their specific career stage. Such mentorship is thus complex, dynamic, and may require a pool of both institutional and extra-institutional mentors.

### Financial pillar.

Formalization of the physician-scientist career path requires dedicated budgetary support that is directly linked to, and supports, their specific activities. The absence of such dedicated support threatens not just the productivity, but fundamental ability, of physician-scientists to recognize and remedy critical problems of unmet clinical need using science in an intentional, focused, and impactful manner. Historical models used to support physician-scientists have increasingly come to depend on the ability of investigators to self-subsidize their research activities through year-to-year adjustments in clinical activity (19, 20). While financially expedient, such models paradoxically create scientific and practical instabilities that jeopardize the very research programs they seek to support. Moreover, such approaches have become increasingly untenable due to simultaneous expansions in the complexity of health care delivery and contractions in clinical margins, while the research funding landscape itself has reached historically competitive heights.

A business model designed to support physician-scientists must provide the appropriate level and duration of support that maximizes the likelihood of establishing and sustaining an extramurally funded research program that can advance discoveries along the translational pathway to clinical impact. Such a model should (a) define and provide an appropriate level and duration of support needed to establish and maintain an extramurally funded research program with monitored output, and (b) not require investigators to cross-subsidize research support with clinical or other administrative sources of support. Core components of such a model include (a) a specific focus on mutually defined and accepted level of research effort and associated compensation, rather than total compensation that encompasses non-research-related effort; (b) a standardized level of expected investigator-based recovery of committed research effort with potential performance-based incentives and defaults; and (c) a standardized multiyear departmental/institutional commitment to supported research effort at all career stages.

A specific focus on research effort–associated compensation, rather than total compensation, serves 2 key purposes. The first is to ensure that financial resources are appropriately aligned with professional activity. This approach helps to clearly define the cost and value of physician-scientist-based research in explicit terms, while also preserving the ability to support non–research-based components of physician-scientist activities through existing clinical and administrative compensation mechanisms. The alternative — a focus on total compensation — creates a competing incentive that discourages increased research efforts in specialties with higher average clinical compensation. The second is to direct the scope of what are often limited resources to the most essential component of their professional activity, their research. A key challenge associated with this approach is the need to establish financially appropriate benchmarks of research compensation that are specific to physician-scientists, and equitable in relation to their area of clinical training and can be measured on a regular basis. One potentially useful resource is the annual AAMC faculty salary report (29), which provides a relative scale of market values across clinical disciplines, faculty rank, geography, and institutional classification. From a broader perspective, adopting compensation benchmarks specific to physician-scientists will require new financial metrics of their value, including potential clinical subspecialty services related to or arising from their research expertise. This approach has the potential to help addresses discrepancies between effort and funding both below and above the NIH cap (30).

The use of a renewable, multiyear commitment to, and expectation of, supported research effort on the part of the institution and investigator serves 3 similarly essential roles. The first is to provide a defined, and more manageable, level, and duration of support and stability for the investigator. The second is to provide a finite and well-defined commitment of resources from the department/institution, with flexibility for sharing across units and over time as well as mechanisms for potential off ramping. The third is to provide investigators with a defined and financially appropriate set of scientific expectations over defined periods of time. The bidirectional nature of such a compact can more broadly promote an alignment of goals and activities between the investigator, department, and institution.

From a budgetary perspective, operationalizing a structured and renewable multiyear approach with clearly defined metrics may initially be achieved by conducting a retrospective budgetary cost accounting to explicitly identify and ringfence already existing resources/mechanisms used to support existing physician-scientists within a given institution. Defining and formally dedicating such resources and mechanisms to physician-scientists can serve as a framework to both (a) enhance the structure, stability and the long-term return on already committed investments that are often made ad hoc on a year-to-year basis, and (b) enable the development of more sustainable cost-resource-balanced business models. Such an approach may more specifically guide the development of new fund-flow mechanisms capable of formalizing ongoing support for physician-scientists and prioritize the direction of existing unrestricted funds toward physician-scientists. Implementation of such a cost-resource-balanced model can additionally make it possible to develop and explicitly quantify new metrics of physician-scientist value, including specific clinical services, such as cell therapies, that arise from their discoveries, and the currently unattributed patient referral volume associated with the reputational stature that physician-scientists bring to academic health systems (31) that may be of particular value in competitive market settings. The proposed formalization of a business model to support physician-scientist research may also help institutions define the specific amount of additional dedicated resources needed to sustain a given cadre of physician-scientists and/or the number of physician-scientists that can be supported with existing resources. The proposed compact between the investigator and institution additionally creates the opportunity to develop professional “off-ramping” mechanisms that can provide structure and flexibility for the faculty member to transition into an alternative career path and for the institution to optimize its return on investment in physician-scientist–based programs both on an individual and strategic level.

### Organizational pillar.

Formalization of the physician-scientist career as a distinct profession ultimately requires intra- and inter-institutional coordination. This need arises from the interconnected and interdependent nature and scope of required scientific, clinical and financial components that often lie beyond the resources of a single academic unit and, in some cases, institution.

On an institutional level, such coordination can be achieved through the creation of a centralized Office of Physician-Scientist Faculty Development. Such centralization is critical to not only marshal and integrate the necessary resources into a cohesive framework of linked academic and financial support but also provide the administrative ability to efficiently adapt to known and unanticipated scientific, financial, and/or personal changes. Centralization can more broadly facilitate the explicit designation and/or strategic reprioritization of existing resources toward physician-scientists in a manner consistent with the institution’s strategic goals for its research enterprise. Such centralization can also facilitate the development and implementation of metrics that can be used to build a physician-scientist–specific business model within and across institutions. Institutional centralization can finally facilitate the development of inter-institutional networks.

The development of inter-institutional networks can broaden the pool of both scientific and clinical expertise and mentorship across fields/disciplines and career stages for individual faculty, and knowledge of different academic and financial mechanisms and models used to support physician-scientists on an organization level; it also fosters the creation of professional networks and/or affinity groups that can generate otherwise unachievable forms of individual and collective benefit (32). Formalizing physician-scientist communities across (and within) institutions may additionally foster collaborations across academic tracks (including academic clinician and non-clinician investigators) by helping to more clearly distinguish their distinct and complementary skillsets.

Core elements of this pillar thus include (a) an institutionally centralized Office of Physician-Scientist Faculty Development (3, 33), (b) inter-institutional networks of physician-scientist faculty, and (c) regular intra- and inter-institutional multipurpose forums to promote scientific and professional exchanges of information and networking of physician-scientists across departments and career stages (32).

The creation of a dedicated institutional Office of Physician-Scientist Faculty Development (33) should specifically address the following considerations. It should first assign/designate physician-scientist faculty with an institutional, rather than department-specific, identity. Second, it should operate as an administratively centralized unit/clearinghouse with the authority to reach across and directly interface with academic departments, administrative and finance units, and the health system and/or physician practice organization, to efficiently track and/or align the necessary resources. Third, it should be equipped with the ability to enable cost sharing across a larger number of units and thereby provide more equity to financially constrained departments. Finally, it should be equipped to function as a specific vehicle through which to interface externally and develop inter-institutional networks.

From an operational perspective, the impact of this Office will only be achieved if staffed with the appropriate combination of stakeholders that not only include physician-scientist faculty across departments, fields, and career stages with varying degrees of leadership/administrative experience and knowledge of institutional culture (33) but also include academic clinicians and administrative staff knowledgeable of and sympathetic to the specific needs and challenges faced by physician-scientists.

## Synthesis, recommendations, and conclusions

In an era of intensifying competition for resources (9, 24) and increasing emphasis on specialization and metric-based accountability that limit opportunities to exploit the dual strengths of the physician-scientist, the need to formalize the physician-scientist profession into an explicit career track has never been greater. Existing models fall short of meeting the professional and financial realities faced by physician-scientists due, in large part, to the continued use of academic and financial paradigms that predate the era of multidisciplinary translational research and compartmentalize clinical medicine and scientific research. This compartmentalization has fostered ad hoc approaches that are both inefficient and unsustainable. Sustaining and strengthening the US physician-scientist workforce demands new models and metrics that accurately capture the unique needs and the distinct value physician-scientists bring to academic medical centers and health systems, such as new clinical programs and reputation-based referrals that are currently untracked. This report proposes one potential framework that is adaptable to different institutions yet unifying across the physician-scientist workforce. Strategic investments in this or comparable frameworks hold the promise of delivering substantial and lasting returns.

Conflicts of interest: ANH reports serving as a consultant for Autobahn Pharma and chairing the MTC Registry Data Monitoring Committee for approved GLP-1 compounds mandated by the FDA funded through United Biosource Corp and supported by Eli Lilly and Novo Nordisk. CC reports research funding from Roche-Genentech, the Department of Defense, Quantum Leap Healthcare Collaborative, and consulting fees from EnliTisa, Novartis, Merck, Aerogen, Boehringer, Healios, Vasomune, Gen1e Life Science, and Matisse.

## Conflict of interest

ANH reports serving as a consultant for Autobahn Pharma and chairing the MTC Registry Data Monitoring Committee for approved GLP-1 compounds mandated by the FDA funded through United Biosource Corp and supported by Eli Lilly and Novo Nordisk. CC reports research funding from Roche-Genentech, DOD, Quantum Leap Healthcare Collaborative, and consulting fees from EnliTisa, Novartis, Merck, Aerogen, Boehringer, Healios, Vasomune, Gen1e Life Science, and Matisse.

## Funding support

This work is the result of NIH funding, in whole or in part, and is subject to the NIH Public Access Policy. Through acceptance of this federal funding, the NIH has been given a right to make the work publicly available in PubMed Central.

- NIH grants T32GM152284, R38HL167237, and P30CA068485 (to CSW).
- VUMC Directorship (to CSW).
- NIH grant R35HL177135 (to CC).
- NIH grants R38HL172261 and R38AI181012 (to EJG).
- NIH grant T32 GM136651 (to BK).
- NIH grants UE5 DK137316, P30 DK123704, and P20 GM120475 (to DCR).
- NIH grant R38 AG070229 (to JMV).
- Burroughs Wellcome Fund Physician-Scientist Institutional Award to Weill Cornell Medicine Physician-Scientist Academy (to KYR).
- NIH grant R38AI174255 (to KYR).
- NIH grants T32HL166134 and R38HL150208 (to DAS).

## Version 1 04/28/2026

In-Press Preview

## Version 2 06/22/2026

Electronic publication
